# Medicinal Qualities of Buttermilk

**Published:** 1881-08

**Authors:** 


### MEDICINAL QUALITIES OF BUTTERMILK.

For a summer beverage there can be nothing more healthy and strengthening than buttermilk. It is excellent for weak or delicate stomachs, and far better as a dinner drink than coffee, tea or water, and, unlike them, does not retard, but rather aids digestion. A celebrated physician once said that if every one knew the value of buttermilk as a drink it would be more freely partaken of by persons who drink so excessively of other beverages; and further compared its effects upon the system to the cleaning out of a cook stove that has been clogged up with ashes that have sifted through, filling up every crevice and crack, saying that the human system is like the stove, and collects and gathers refuse matter that can in no way be exterminated from the system so effectually as by drinking buttermilk. It is also a specific remedy for indigestion, soothes and quiets the nerves, and is very somnolent to those who are troubled with sleeplessness.

There is something strange in the fact that persons who are fond of buttermilk never tire of singing its praises, while those who are not fond of it never weary of wondering how some people can drink it. So far as is possible, people should overcome their aversion to it, and learn to drink it for health's sake. One gentleman of our acquaintance is so extremely fond of it that we knew him one time to drink about three glasses, then set his glass down with a thud, exclaiming earnestly as he smacked his lips: "That's food and raiment both;" while another buttermilk enthusiast made the statement once that where the liver has become lifeless from torpidity and inaction, and is too dead to perform its functions, buttermilk will cause a new one to grow in. Whatever exaggerated statements may have been made concerning buttermilk, its medical properties cannot be overrated, and it should be more freely used by all who can get it. Every one who values good health should drink buttermilk every day in warm weather, and let tea, coffee and water alone.

For the benefit of those who are not already aware of it, I may add, that in the churning the first process of digestion is gone through, making it one of the easiest and quickest of all things to digest.

It makes gastric juice and contains properties that readily assimilate with it, with little or no wear upon the digestive organs.

